# Posterior vault “free-floating” bone flap: indications, technique, advantages, and drawbacks

**DOI:** 10.1007/s00381-021-05281-x

**Published:** 2021-07-15

**Authors:** Gianpiero Tamburrini, Martina Offi, Luca Massimi, Paolo Frassanito, Federico Bianchi

**Affiliations:** Pediatric Neurosurgery, Institute of Neurosurgery, Fondazione Policlinico Gemelli, IRCCS, Università Cattolica del Sacro Cuore, Rome, Italy

**Keywords:** Craniotomy, Free bone flap, Posterior vault, Syndromic craniosynostosis

## Abstract

**Background:**

The enlargement of the posterior cranial fossa volume is considered one of the main steps of the surgical management of children with multiple sutures craniosynostosis. Different management options have been proposed including fixed expansive craniotomy, free bone flap craniotomy, and distraction osteogenesis.

**Objectives:**

To review indications to “free bone flap” craniotomy for the posterior fossa expansion, detailing advantages, disadvantages, and complications related to the technique.

**Results and conclusions:**

A review of the literature shows that “free bone flap” posterior expansion cranioplasty still has a role, particularly in infants with thin and “honeycomb” structure of the bone, allowing to gain adequate intracranial volume increases and to postpone to a more adequate time surgery aimed at anterior cranial fossa expansion.

## Indications and rationale

Posterior cranial fossa engorgement is a known distinct anatomic feature of children with syndromic craniosynostosis. Progressive skull base synchondrosis and the fusion of the lambdoid sutures are the main actors of this phenomenon leading to an early volume reduction of the posterior cranial fossa [1–5]. Stenosis of the jugular foramina, as well as the low-lying position and direct constriction of the sagittal sinus and torcula, determines a venous hypertension, which, together with posterior fossa craniocerebral disproportion, is the background for tonsillar herniation and hydrocephalus development [1–5]. It has been extensively demonstrated that the rate and rapidity of fusion phenomena is different in different syndromes, being documented in 50% of children with Pfeiffer syndrome, 70% of patients with Crouzon syndrome and of those with oxycephaly and 100% of children with Kleeblattschadel [5]. When this process is already active at birth, the cranial vault might acquire a progressive mold deformity shaped in a “honeycomb” appearance secondary to the different fusion timing. In this contest, the need for posterior cranial fossa expansion conflict with an extremely fragile bone structure [6, 7].

The latter definitely limits the possibility to consider both a fixed cranial expansion as well as bone distraction (external as well as internal distractors and/or springs) [8, 9]. The increased intracranial pressure might, on the other side, be positively harnessed to drive occipital and sub-occipital bone structures expansion once relieved from their attachment to the adjacent biparietal region, which is the main rationale and absolute indication for the “free-floating” technique. A relative indication, which needs a comparison with the other named surgical possibilities (fixed expansion and bone distraction), is represented by all other conditions of bilambdoid fusion with a relatively preserved bone structure [10, 11].

## Technique

**Fig. 1:**
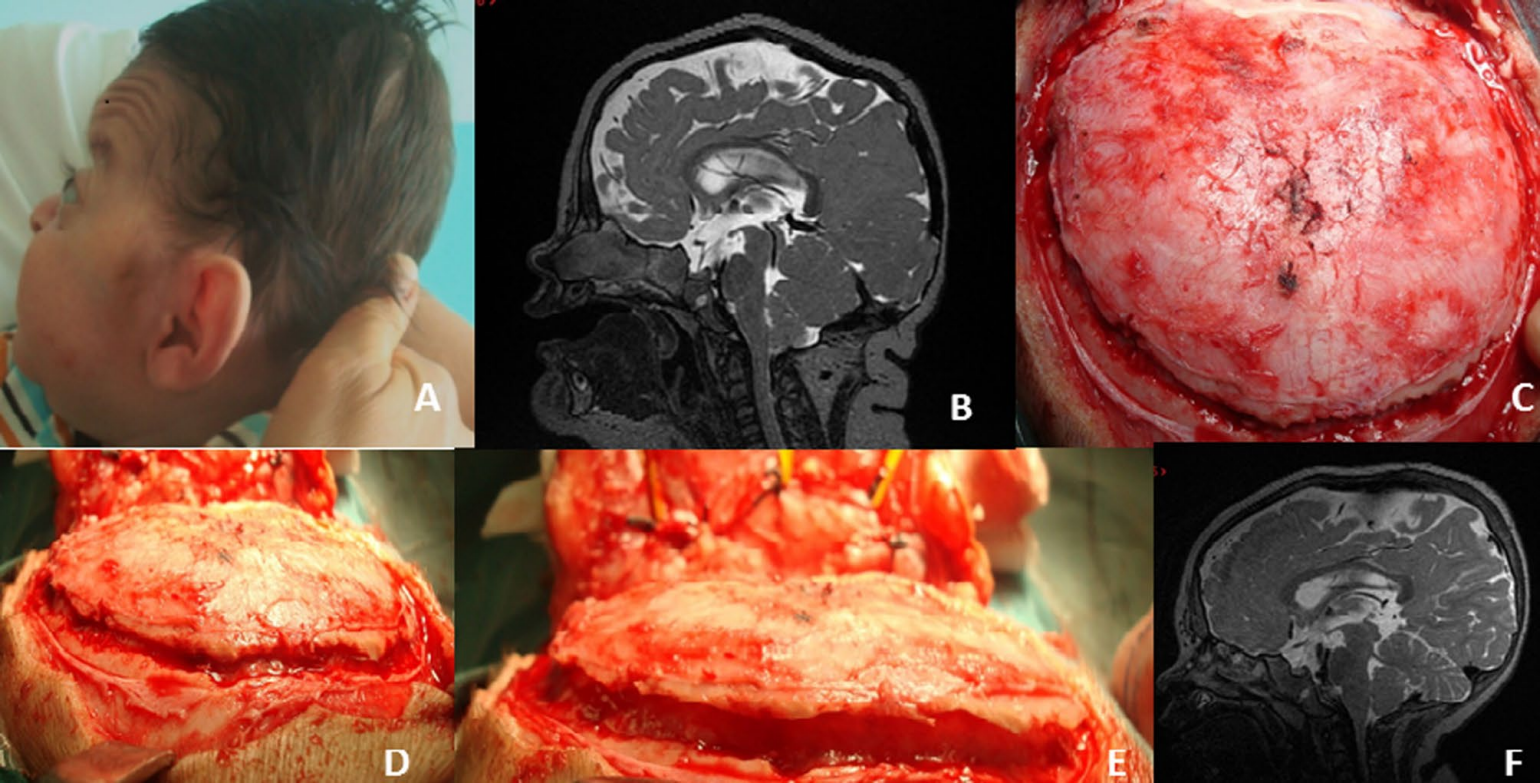
Six-month-old child with Apert syndrome. **A** Lateral view of the child documenting a marked occipital flattening of the head. **B** Preoperative T2 sagittal MR image documenting a constriction of the posterior cranial fossa structures and a low lying torcular. **C** Intraoperative view of the exposed occipital region before completing the craniectomy and detachment of the bone from the underlying dura. **D** Intraoperative view after completing the craniectomy and dissection of the occipital bone from the dura. **E** Intraoperative view before closure of the skin flap demonstrating the early volume gain of the cerebral structures. **F** Postperative T2 sagittal MR image documenting the expansion of the posterior cranial fossa structures

**Fig. 2:**
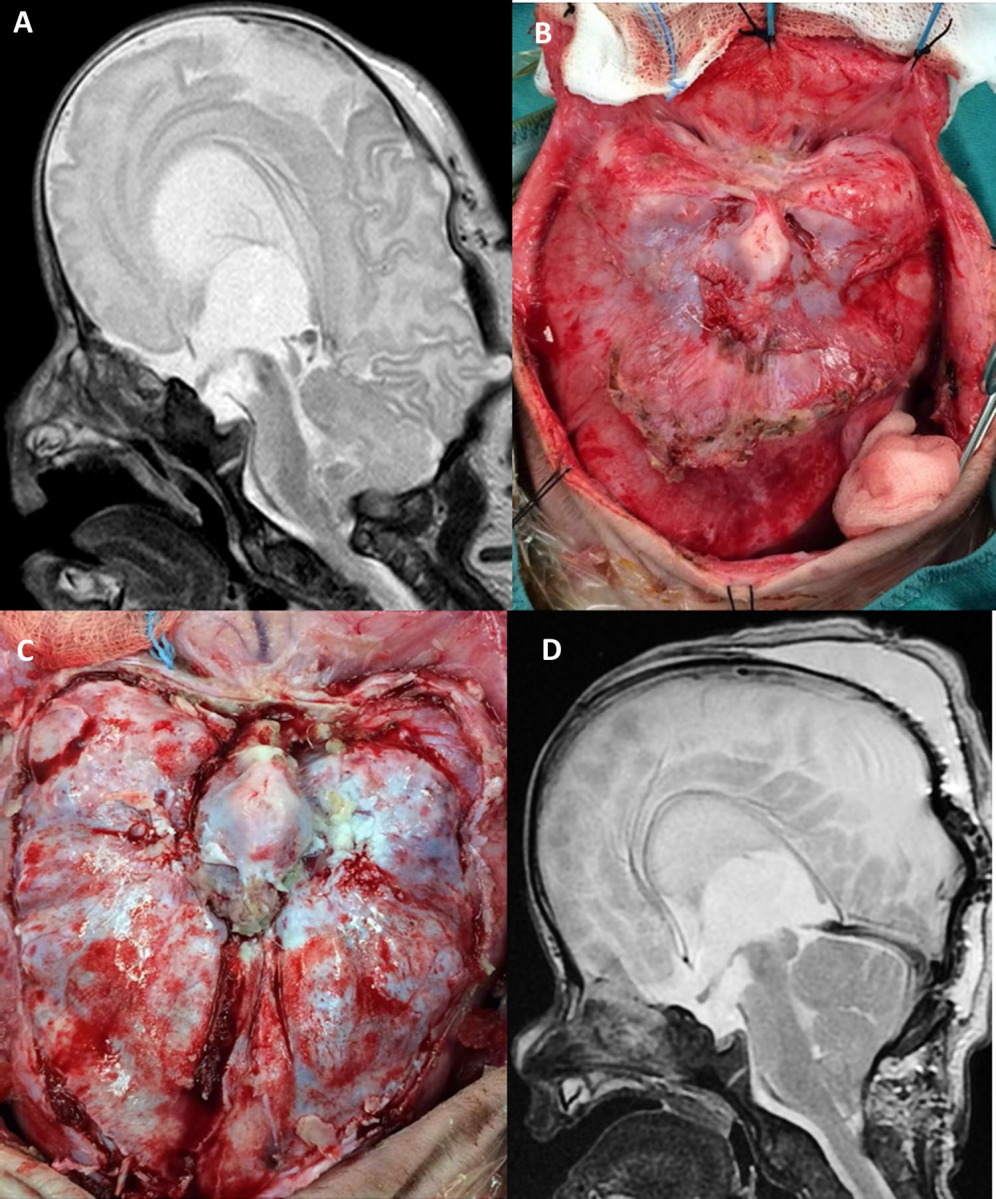
**A** Two-month-old child with Crouzon syndrome. **B** Preoperative sagittal T2 MR view documenting the presence of hydrocephalus and severe constriction of the posterior cranial fossa cerebral and venous structures. Multiple sites of brain herniation through the bone are also visible. **C** Intraoperative view after the exposure of the occipital region: the bone is extremely irregular and thinned. **D**-**E** Intraoperative view after biparted parietooccipital bone craniectomy and bone detachment. **F** Postoperative sagittal T2 MR view demonstrating an improvement of the posterior cranial fossa volume and an associated reduction of the ventricular size

The child is positioned in prone position covered with a cotton infant head holder to reduce the risks of skin erosion and eyes appropriately distanced. A posterior bilambdoid skin incision is performed with extension to the biparietal region at the level of the cranial vault. In the case of children with a honeycomb structure of the bone, the existing holes are used as a pathway to release the bone from the dura in a circular way with lower limit below the transverse and sigmoid sinuses. In patients with more compact bone structure, two parietal drill burr holes are performed 1 cm from the midline and two further burr holes are performed at the level of the lateral third of the transverse sinus. Pediatric craniotome saw is used to join the two paramedian parietal burr holes with the burr holes at the level of the transverse sinus, whereas piezosurgery (Piezosurgery®, Mectron) is used to cut the bone at the level of the midline parietal bone structure as well as at the inferior border of the craniotomy, which stays 2 cm below the transverse/torcula site. A dural dissector is hence used to detach the bone from the dura mater from the lateral border of the craniotomy up to the midline where the sagittal sinus is left in place as an anchor point for the bone. Similarly, the lower border of the craniotomy is detached from below up to the torcula and laterally up to the midline. Superficial subcutaneous and cutaneous layer are closed after accurate hemostasis with no subcutaneous drainage left in place (Fig. 1). In infants with marked venous hypertension, the flap can be divided in two in order to protect the sagittal sinus and/or the torcula avoiding the risk of transosseous vein damage (Fig. 2).

## Review of the literature

In order to better define the “free-float” technique, a review of the English pertinent literature was performed to compare the existing procedures with the hereby reported one. A comprehensive research was carried on using PubMed and the subsequent keywords: (1) syndromic craniosynostosis and posterior vault expansion, (2) syndromic craniosynostosis and posterior cranial fossa. From the aforementioned analysis, 48 papers were found but only 6 dealt specifically with the surgical techniques. In the following paragraph will be reported the results of the revision.

## Results

There are few series in the literature that selectively deal with posterior free-floating cranial vault expansion in the management of syndromic craniosynostosis. The first historical report dates back to 1996. Sgouros et al. reported the results of 22 children, 16 of whom affected by multiple sutures synostosis, with a defined syndrome in 13 of them, who underwent posterior expansion with a “free floating” occipital bone flap, using two different surgical techniques, a single bone flap extended 2 cm. above the lambdoid sutures with lower limit at the torcula sigmoid sinuses junction or a biparted double bone flap with a midline bone strip craniectomy along the lower third of the sagittal sinus. The procedure entered in the multi-staged management protocol of these patients, allowing, as first surgical procedure, to postpone fronto-orbital advancement in 10/22 cases (45.4%) and avoid it in 3/22 cases (13.6%) [12, 13]. Few years later, Cinalli et al. reported four cases (two with Crouzon’s syndrome, one with Kleeblattschadel, and one with complex craniosynostosis) presenting multiple-suture synostosis with severe occipital flattening, posterior fingerprint impressions, and chronic tonsillar herniation who underwent occipital vault remodeling and sub occipital bone decompression. The technique proposed by the authors differs from a free-floating cranial vault expansion in the sense that the occipital bone was completely detached and mechanically expanded posteriorly. Immediate results were reported to be satisfactory both in terms of volume obtained as well as correction of the tonsillar herniation. Recurrence was, however, reported in one child 15 months after surgery [2]. Most of the other data refer to series comparing conventional osteotomy with distraction-based surgical procedures using external distractors or springs. Taylor et al. compared posterior cranial vault conventional osteotomy with distraction osteogenesis in an overall population of 25 children 16 undergoing conventional osteotomy and 9 distraction osteogeneses. They did not find significant differences in terms of median length of surgery, intra- and postoperative blood loss, intensive care unit postoperative stay, and postoperative complications. However, in this paper, the populations considered were quite different. Eight of the nine children who underwent distraction osteogenesis were indeed affected by syndromic craniosynostosis (88.9%) whereas only three of the fifteen children (20%) who underwent conventional posterior osteotomy had multiple sutures synostosis. These differences might certainly have influenced the results in terms of blood loss and length of surgery that have been reported by the authors. Of interest, four (44%) of the distraction osteogenesis patients had undergone previous cranial vault surgery, consisting of four anterior cranial vault surgeries and one posterior remodeling, whereas six of the conventional osteotomy patients (38%) had undergone previous cranial surgery, consisting of four sagittal synostectomies, one anterior cranial vault remodeling, and one posterior remodeling [14]. In the series of Nowinski et al. volumetric data for three different surgical techniques used for the management of posterior cranial vault constriction were compared. On a total number of 6 children, two cases were treated with posterior cranial vault expansion by free-floating parietooccipital bone flap, two with springs assisted, and two with internal distractors-assisted expansion. The volumetric analysis showed an expansion of 13 and 24% for the free posterior flap, 18 and 25% for the trans lambdoid springs, and 22 and 29% for the distractors, actually demonstrating the absence of significant differences in terms of volume gain among the three different kinds of procedures. No data were reported concerning the follow-up, actually limiting the possibilities of fully compare advantages and disadvantages of the three different surgical procedures [10]. Sprujit et al. came to similar conclusions comparing the results in terms of volume gain obtained in 10 children who underwent conventional posterior fossa enlargement with those obtained in 9 children who underwent springs assisted posterior calvarial expansion. Comparable results were reported also in terms of correction of preoperative papilledema, correction of secondary tonsillar herniation and time for recurrence (mean age at recurrence/need of reoperation = 3.5 years) [15].

## Advantages and complications

The main advantage of the “free-floating” occipital expansion resides in the possibility to use it in the most unfavorable conditions (tiny honey-comb internal bone structure, severe constriction of intracranial structures). Its potential efficacy stays, in fact, that it gives to the constricted brain the possibility to become the first actor for its volume gain. Moreover, being based on a simple release of the closed sutures blood loss is limited [10, 12, 13, 15].

Being a hardware-independent operation, there is no need to undergo a secondary surgical procedure as it happens when springs or internal distractors are used. Similarly, to what it has been proposed for the coronal ring “free floating” posterior expansion may be also useful to prepare the hardware positioning, which can be postponed at a time the bone structure become able to better sustain them, thanks to its thickening once released from the chronic increase of the intracranial pressure [8].

One of the disadvantages of this technique is that freeing the bone without subsequently fixing it, means the need to avoid a purely supine position at least in the first 30–40 days after surgery, for the related risk to counteract the brain driven posterior cranial expansion. Concerning the intraoperative phase, it can be argued that skin closure might be more difficult once having detached and freed the bone compared with its simple freeing that is what is required for springs or distractors positioning. Also, the volume amount potentially obtained with distraction osteogenesis is higher as it has been reported by some authors [10, 15], though this advantage has not been universally demonstrated [14]. During the follow-up, the most claimed disadvantages of the “free floating” technique are the risk of relapse and incomplete ossification. Although these last are a logical extension of observed complications, they are poorly defined and incompletely quantified in the literature. In addition, it is quite unexpected to observe a lack of ossification in patients whose disease is related to increased bone development making the “free-floating” technique ideal for them. [15].

## Conclusions

In the era of the broadening spectrum of indications for distraction osteogenesis, “free-floating” posterior cranial fossa expansion still plays a role as a measure devoted to gain space for vital structures when we do have a relevant constriction of the posterior fossa already present at birth and/or rapidly developing during the first months of life. In this specific population of infants, the procedure takes advantage from the increased intracranial pressure to enlarge, at least temporarily, the intracranial volume, without the need to implant any device, with limited blood loss. As for the natural history of the main candidates to this procedure, it is not a definitive measure, but only one of the wide spectrum that are frequently needed in this context, able to gain time for the correct planning of more definitive procedures.
